# Efficient Blind Spectral Unmixing of Fluorescently Labeled Samples Using Multi-Layer Non-Negative Matrix Factorization

**DOI:** 10.1371/journal.pone.0078504

**Published:** 2013-11-08

**Authors:** Thomas Pengo, Arrate Muñoz-Barrutia, Isabel Zudaire, Carlos Ortiz-de-Solorzano

**Affiliations:** 1 Centre for Genomic Regulation, Barcelona, Spain; 2 Cancer Imaging Laboratory, Center for Applied Medical Research, University of Navarra, Pamplona, Navarra, Spain; 3 Biomarkers Laboratory, Center for Applied Medical Research, University of Navarra, Pamplona, Navarra, Spain; Institute of Psychology, Chinese Academy of Sciences, China

## Abstract

The ample variety of labeling dyes and staining methods available in fluorescence microscopy has enabled biologists to advance in the understanding of living organisms at cellular and molecular level. When two or more fluorescent dyes are used in the same preparation, or one dye is used in the presence of autofluorescence, the separation of the fluorescent emissions can become problematic. Various approaches have been recently proposed to solve this problem. Among them, blind non-negative matrix factorization is gaining interest since it requires little assumptions about the spectra and concentration of the fluorochromes. In this paper, we propose a novel algorithm for blind spectral separation that addresses some of the shortcomings of existing solutions: namely, their dependency on the initialization and their slow convergence. We apply this new algorithm to two relevant problems in fluorescence microscopy: autofluorescence elimination and spectral unmixing of multi-labeled samples. Our results show that our new algorithm performs well when compared with the state-of-the-art approaches for a much faster implementation.

## Introduction

The ability to fluorescently label specific cell structures has improved our understanding of the mechanisms that govern living organisms. In this paper, we address two related issues that complicate the analysis of fluorescently labeled samples: the detection of fluorescent objects in the presence of autofluorescent material and the spectral unmixing of fluorescent channels in samples labeled with multiple fluorochromes.

The first issue has to do with the elimination of strongly autofluorescent material that can be easily mistaken for real fluorescence staining. Autofluorescence comes from different sources: some extracellular components (e.g., collagen fibers) and cell types are highly autofluorescent (e.g., eritrocites or macrophages [1]). It can be biochemically reduced using autofluorescence quenching agents such as trypan blue or crystal violet [2], at the expense of spurious emissions at longer wavelengths [3]. Other techniques used in flow cytometry exploit the different lifetime (i.e., fluorescence-lifetime imaging microscopy, FLIM) between autofluorescence and fluorochrome staining [4], [5]. However, measuring FLIM differences requires specialized hardware [6], [7]. Billington’s review [8] describes other chemical and biological methods to reduce autofluorescence and image acquisition hardware to help in the discrimination between the autofluorescence and the fluorescence of interest. We, following Ecker *et al.* [9] and others, treat autofluorescence as an additional fluorochrome in the samples, and removing it is dealt with as a spectral unmixing problem.

The second issue deals with the non-ideal nature of fluorescence spectra and optical filters. In a biological sample, the fluorescence emission of each dye is usually recorded using an appropriate combination of excitation and emission filters. In a fluorescence microscope, a specific filter cube is required for each fluorescent dye. Each filter cube has an excitation filter, a dichroic mirror and an emission filter. The emission filter for a particular fluorescent dye is designed to include the nominal emission wavelength for its reference spectrum, and a bandwidth that implements a compromise between fluorochrome specificity and intensity. The fluorochrome emission spectrum gradually decays towards the longer wavelengths and sharply towards the shorter ones [10], [11]. This gradual decay in the emission spectrum causes the signals from fluorescent dyes to mix with the emissions of longer wavelength fluorochromes. Spectral unmixing is normally used to separate each original signal from the recorded mixtures.

Several methods for spectrally unmixing fluorescent emissions have been proposed in the literature. Zimmermann [10] was among the first to model the recorded fluorescence emission as a linear combination of the reference spectra of all involved fluorochromes, thus adopting the Linear Mixing Model (LMM) previously used in chemistry [12]. When the linear combination coefficients –also called crosstalk coefficients- are known, the simplest method to separate the spectral components of the mixture is a weighted subtraction of the intensities of the longer wavelength channels from the intensities of the shorter ones [10]. The underlying assumption is that a fluorophore emits only at its nominal or longer emission wavelengths. Under this assumption, the cross-talk coefficients can be estimated using linear regression on the joint histogram [11]. Strictly speaking this assumption does not hold true because there is always fluorescence emission below the nominal emission wavelength. Furthermore, this method requires having at least one channel where the detected intensities come from a single fluorophore. On the bright side, the model is simple, and provides acceptable results in many practical settings.

If the model is extended by allowing contributions from longer wavelength emitting dyes, the problem cannot be solved by subtraction, and a full system of equations must be solved. The method was first proposed by Castleman [13] for the color compensation of multicolor FISH images. The basic principle is as follows: for a given pixel, the known terms of the equations are the detected emissions in all detection channels and the relative contributions of the pure fluorochromes to each detection channel. The unknowns to be calculated are the specific contributions of each fluorochrome to the intensity of each voxel. The relative contribution of each pure fluorochrome to each detection channel can be measured *a priori* in single-stained samples, or on areas of multiple-stained samples that contain only one of the fluorochromes. To solve the system of equations the standard method uses quadratic error minimization [12], [14]. To better fit the solution, all fluorochrome contributions are constrained to be non-negative, thus the solution can be calculated using a Non-Negative Least Squares (NNLS) algorithm [15].

In general, it is difficult to correctly estimate the relative contributions of a fluorophore to each detection channel, since they depend not only on the spectra of the fluorophores, but also on other factors, such as their concentrations, quantum efficiency, photobleaching rates or illumination conditions. In other words, the spectral characterization of the fluorophores is time consuming and not fully reliable as the spectra are subject to experimental and biological variability. Moreover, the task may also require additional hardware and can be time consuming. Consequently, methods that simultaneously estimate both the relative contributions of the fluorochrome to each channel and the actual fluorochrome intensities per voxel –i.e., blind methods– have been recently proposed. Among these methods, derived from Statistics and Linear Algebra [11], [16], [17], Non-Negative Matrix Factorization (NMF) has emerged as one particularly successful technique. It will be introduced in the next section. Worth mentioning is also the family of Parallel Factor Analysis (PARAFAC) [18] or Non-negative Tensor Factorization (NTF) [19], [23]. These methods include the absorption spectrum of fluorophores in the analysis, since the intensity of the light emitted by a fluorophore does not only depend on the emission spectrum but also on their absorption spectrum. Therefore, whenever we excite a mixture of fluorophores with a sequence of excitations wavelengths, the light detected by a fixed array of emission channels will be different for each fluorophore. By acquiring all channels for each excitation wavelength, we have additional information, which can be used to recover the correct fluorophore distribution. The downside of the method is the added complexity of acquiring all possible combinations of excitation and emission wavelengths. Another interesting approach was proposed by Gavrilovic *et al.* [20]. In their method, the cross-talk is estimated from the shift in the spectral angles computed from the pixel hues. This spectral phasor analysis is a promising technique recently capturing attention in the literature [21], [22].

The use of NMF for the spectral unmixing of fluorescent images was first proposed by us [23] and Neher *et al*. [24]. Later, Woolfe *et al*. [25] applied NMF to remove autofluorescence from images using a two-step method (i.e., comparing images before and after staining). Here, we build on our initial work [23] to develop a novel algorithm that overcomes the limitations of previously published methods, namely their slow and suboptimal convergence, especially when the fluorochrome spectral emissions strongly overlap. Furthermore, we propose a novel initialization for the crosstalk coefficients that fits the typical spectral emission of the fluorochromes more closely than the shifted Gaussian approximation used by Neher *et al.* [24]. These methodological improvements allow us to successfully apply the NMF algorithm to two very relevant tasks: the detection of fluorescently labeled nuclei from autofluorescent material and the spectral separation of the emissions in multiple fluorescence in-situ hybridization samples (M-FISH). Removing autofluorescent material is performed in one step –one single acquisition- using the additional information given by extra channels -as described by Roederer *et al.* [26] and followed by us [27]- which results in a simpler approach than the two step method of Woolfe *et al*. [25].

The paper is organized as follows: in the next section, we introduce the basic theory and the existing solutions for NMF. Then, we explain the limitations of the existing methods and how we have addressed them in our implementation. In the Materials and Methods section, we present the experiments and materials used to test our algorithm. Finally, the results are presented and discussed.

## Materials and Methods

### Ethical Statement

The samples used in this study were obtained, after the subject’s consent, within the context of the project “Application of the FICTION technique as a diagnostic tool in lung cancer detection”, granted by the Health Division of the Government of Navarra. The protocol was approved by the Committee on the Ethics of Research of the University Clinic of the Faculty of Medicine of the University of Navarra. Written informed consent was obtained from each subject.

### Linear Mixing Model

The basic premise of linear mixture modeling is that a given microscope field-of-view contains a known number of fluorochromes with relatively constant spectral properties. As the emitted light comes from the excitation of the whole field-of-view, the emissions of the different fluorochromes contribute to the intensities detected per channel in an additive way. Moreover, the overall gain with which a probe contributes to this addition is proportional to its contribution at each voxel.

Following the LMM theory [12], the intensity measured in the emission channel *i* at a pixel *j* (*y*_ij_) is a linear combination of the contributions of each fluorochrome *k* (*h*_kj_) weighted by the crosstalk of the fluorochromes into that channel (*a*_ik_). These crosstalk coefficients are non-negative values defined between 0 and 1. In matrix form, we can express the resulting system of equations as **Y** = **AH**, where **Y** is the *L*×*N* matrix of detected intensities, being *L* the number of spectral channels and *N* the number of pixels, arranged as columns; **A** is the *L*×*M* crosstalk coefficient matrix, where *M* is the number of fluorochromes used. Finally, **H** is the *M*×*N* fluorochrome emission matrix. The goal of a spectral unmixing algorithm is to find the real contributions (**H**) of the fluorochromes to the voxel intensities (**Y**) measured in each channel.

### Non-negative Matrix Factorization

Solving a NMF problem, previously referred to as Non-negative Matrix Approximation [28] or Positive Matrix Factorization [29], can be formulated as the following optimization problem: 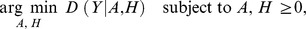 where the function **D** measures the goodness-of-fit of the estimation of **Y**, given **A** and **H**. A number of functions **D** have been proposed [28], [30], being the two most common the L_2_-norm and the Kullback-Leibler divergence [31]. The first assumes the presence of additive Gaussian noise in the measurements, while the second assumes Poisson distribution [32]. Lee and Seung [32] proposed a solution for both scenarios that uses a gradient descent algorithm with multiplicative update rules. The algorithm starts from an initial estimation of both **A** and **H** and then moves along the direction of maximum descent of the objective function, by alternatively updating **A** and **H**. The use of these multiplicative updates guarantees maintaining the positivity of both matrices.

In general, a unique solution cannot be guaranteed [33], [34], [35] since for any solution of the NMF algorithm, infinite alternative solutions may be constructed. Namely, if **B** is an invertible matrix, i.e. **BB^−1^** = **I**, the following matrices **A′** = **AB** and **H′** = **B^−1^H**, are also solutions of the optimization [33], [34]. To constrain the problem, a regularization term can be added to the objective function. This term is designed to improve the convexity of the distance function, which in turn reduces the number of local minima. A fair number of regularization functions have been proposed in the literature and the corresponding update rules have been calculated for many of them (see [28], [31], [36], [37] for a review). Common regularization terms are the L_2_-norm, the L_1_-norm [38] or the ratio between both presented by Hoyer *et al.* [39]. We adopt the latter as it gives a preference to spatially sparse solutions with maximal spectral overlap. We will refer to it as NMF with *Segregation Bias* (NMF-SB).

### Shortcomings of the Existing Methods and Solution Proposed

Two common, unsolved problems of NMF are the existence of local minima in the objective function and the slow convergence of the multiplicative rules used by the gradient descent algorithm.

Due to the existence of local minima, the final solution strongly depends on the initialization of the algorithm. Neher *et al.* [24] chose to initialize **A** using Gaussian functions centered at the nominal wavelengths of the reference spectra. Each Gaussian spectrum occupied a column in **A**. This Gaussian approximation is not well suited in most settings, because it does not take into account the asymmetry of the typical fluorochrome spectra. To compensate for this, they proposed to shift the Gaussian spectra towards the longer wavelengths: by shifting the Gaussian spectrum, its center of mass is shifted closer to the center of mass of a real asymmetric spectrum (see supplementary material of [24]). Instead, we propose to use an exponential initialization matrix.

To address the slow convergence of the algorithm, we based our proposal, called multi-layer NMF (NMF-ML) on the technique developed by Cichocki *et al.* [40]. The main idea behind the method is to apply NMF to the matrix components resulting from a previous NMF decomposition. Therefore, once **A** ^(1)^ and **H** ^(1)^ are obtained from a first NMF calculation, **H** ^(1)^ is further decomposed into **A** ^(2)^ and **H** ^(2)^ by applying the NMF decomposition again. The result is the decomposition of **Y** into **A** ^(1)^ **A** ^(2)^ **H** ^(2)^ and so on. In essence, the basic matrix **A** is replaced by a set of cascade (factor) matrices. Since the model is linear, all the matrices can be merged into a single matrix **A**, if no special constraints are imposed upon the individual matrices **A^(n)^**. The global convergence criteria at the n^th^ decomposition can be related to the distance between **A** and the identity matrix, D(**A**,**I**) since at convergence, **H** ^(n−1)^ = **A** ^(n)^ **H** ^(n)^, which in turn implies that **A** ^(n)^ = **I** or else, D(**A**,**I**) = 0. Although a theoretical framework for the analysis of multi-layering is still an open question, intuitively it accelerates convergence and alleviates local minima by distributing the structure of the A matrix [41].

Next, we describe the initialization of the crosstalk coefficient matrix **A** and the fluorochrome emission matrix **H**, present the implementation of our novel NMF-ML algorithm, and test it in our two target applications.

#### Matrix initialization

To initialize the crosstalk coefficient matrix **A_0_**, we build on the following observations: i. all fluorochrome emissions are more intense in their nominal emission channel than in the other channels; ii. all fluorochrome contributions are strictly positive; iii. the spectrum of any fluorophore –the columns of the mixing matrix **A**- falls gradually towards longer wavelengths and sharply towards the shorter ones. In summary, assuming that the rows of **Y** and **H** are ordered by increasing wavelength, the matrix form of **A** requires a diagonally dominant non-negative matrix, where the lower triangular part of the matrix contains higher values than the upper triangular part.

To fulfill these requirements, our proposal is to initialize **A** as an exponential matrix. If L = M, each element of the lower triangular part and the diagonal is initialized as **a**_i,j_ = 2^−(i–j)^, while the upper triangular part is initialized as **a**_i,j_ = 2^−L^ To ensure that the sum of the components for each pixel does not change after the unmixing i.e., that the sum of each column of **Y** equals the sum of each column of **H**, the sum of each column of **A** is normalized to one. An example of a 3×3 matrix (before normalization) is:
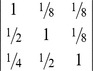



If the matrix we wish to generate is not square (*L≠M*), we first create a squared matrix of size equal to the largest dimension and then, interpolate it to the desired size.

To initialize the fluorochrome contribution matrix **H**, we make it equal to the detected intensity matrix **Y**, i.e. **H** = **Y** if *L = M.* Otherwise we use random values.

#### Proposed NMF-ML algorithm

After initialization, **H** and **A** are alternatively updated using the multiplicative update rules described in [40]. The values of α_1_ and α_2_ enforce the sparseness of the H and A matrices respectively and are typically in the range of 0.001–0.5 [40]. In our experiments, they were fixed to 0.01 and 0.1 respectively. Negative values are clipped to a small value **ε** = 10^6^ times the machine precision, as used in NMFLAB [39]. At the 20^th^ iteration the first layer of the NMF algorithm is created: the cumulative matrix **A_c_** is initialized to the current estimation of **A**, and **A** is reset to an *M*×*M* exponential matrix. The fluorochrome emission matrix **H** is calculated using the regularized Moore-Penrose inverse, setting all negative components to **ε**. Both matrices **A** and **H** are then updated until the 39^th^ iteration. At the 40^th^ iteration and every 20 iterations onwards, a new NMF layer is created: **A_c_** is updated by multiplying it by the current estimation of **A** and then, the **A** matrix is reset. This way, at any given time **A_c_** contains the cumulative product of all the **A** matrices. The fluorochrome emission matrix **H** is calculated using the regularized pseudo-inverse of **A_c_**. Upon convergence, the **A** matrix approaches the identity matrix **I**.

After convergence, the final **H** matrix is calculated using the **A_c_** matrix using the same regularized Moore-Penrose inverse used every 20 iterations, setting all negative components to 0. Finally, the **A_c_** matrix is returned along with **H**. The following pseudo-code describes the proposed NMF-ML algorithm:

### Detection of Stained Nuclei in the Presence of Autofluorescence

In this application, the NMF-ML algorithm is used to distinguish autofluorescent cell nuclei from single-color stained fluorescence nuclei in microscopy images. The underlying idea is that autofluorescence can be treated as any other fluorochrome, and distinguished from actual fluorochrome emissions by its extended spectrum. Therefore, we feed the algorithm with pixel data from three input channels –wide enough as to cover the broad fluorescent emission of autofluorescence– and force the output to belong to one of two classes: single-channel fluorescence –corresponding to stained nuclei- or autofluorescence –corresponding to autofluorescent objects-. We first test the performance of the algorithm on artificially generated images, and then apply it to real images.

#### Synthetic images

**Figure 1:**
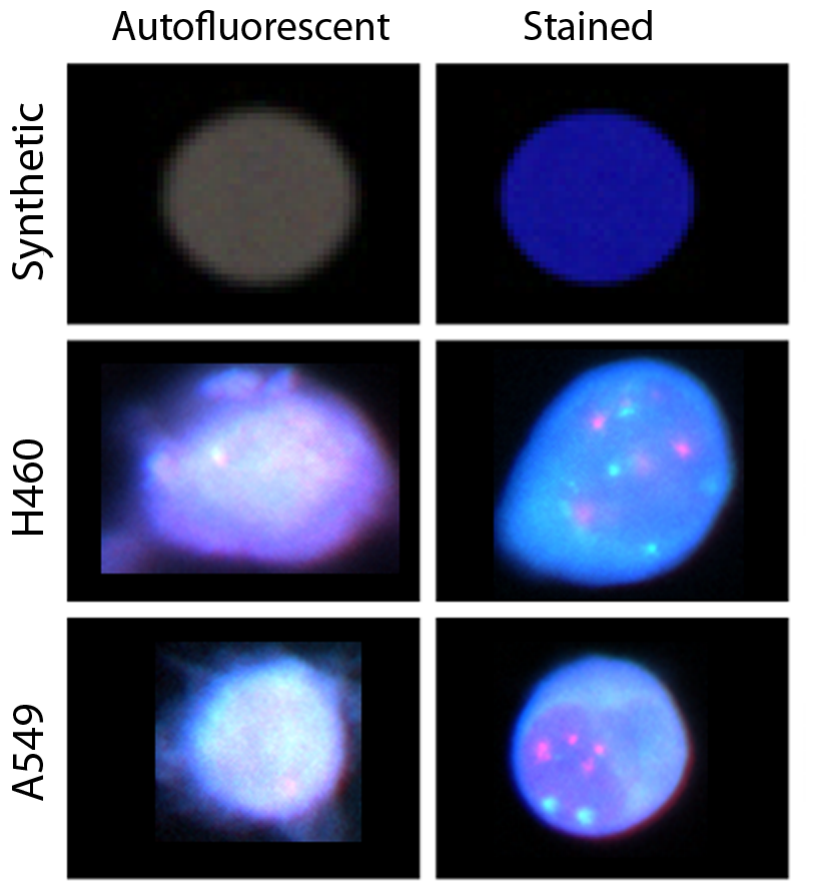
Examples from the image datasets. The left column shows autofluorescent objects. The right column shows stained objects. The three rows represent the three types of samples used: the synthetic object (top row), H460 cell line (middle row) and the A549 cell line (bottom row).

We created 100 synthetic 256×256, 3-channel images containing four round objects simulating cell nuclei. Two nuclei displayed mild autofluorescence, one strong autofluorescence and one simulated a stained nucleus, with a strong emission in the blue channel. Autofluorescent nuclei are assigned similar intensities in all channels (see Figure 1).

To create each image, there are ten parameters that need to be selected:

- emission intensity of the strong autofluorescent nucleus (1 parameter),
- emission intensity of the two mild autofluorescent nuclei (1),
- emission intensities of the three spectral channels of the stained nucleus (3),
- transmittance of the emission filters corresponding to the three acquisition channels (3),
- amount of additive noise (1),
- amount of multiplicative noise level (1).

Each parameter is assigned an admissible interval of values. If we consider the parameter space as the cross product of all admissible intervals for each parameter, the result is a 10-dimensional space where each point represents a particular combination of parameters. To choose the set of parameter combinations –i.e., the points in the 10-dimensional space– with a high degree of uniformity, we generated the first 100 points from a 10-dimensional version of a Halton point set [42], [43], which is specially designed to generate any number of points uniformly distributed in any number of dimensions.

#### Real images: sample preparation and image acquisition

Broncho-alveolar lavage samples (BAL) [44] from asymptomatic subjects were mixed with cells from two established lung cancer cell lines, A549 and H460. The samples were stained with a nuclear immuno-marker antibody against the protein hnRNPA1, conjugated to Alexa 350. This protein is preferentially expressed in the nuclei of lung cancer cells. Therefore, the samples should contain cancer cells with positive hnRNPA1 stained nuclei, normal epithelial cells with low hnRNPA1 protein content, highly autofluorescent macrophages, and –usually autofluorescent– organic debris. All images were acquired using a Zeiss Axioplan2ie microscope (Wetzlar, Germany) with a 20×0.75 NA Plan-Apochromat objective. At each field of view, we acquired three images using the following filter cubes: BLUE (excitation filter 365 nm, dichroic 395 nm, emission filter center 445 nm, bandwidth 50 nm), AQUA (ex. 436/20, dic. 455, em. 480/40) and RED (ex. 546/12, dic. 560, em. 607/65). All images were acquired using in-house developed software [45] that controls the microscope, and a cooled CCD camera Photometrics CoolSNAP (Roper Scientific, Tucson, Arizona, USA). All algorithms were programmed in the MATLAB programming environment (Mathworks, Natick, MA, USA). Image processing was performed using the DipImage toolbox (TUDelft, Delft, The Netherlands). The statistical analysis was performed using the open-source software R (The R Foundation for Statistical Computing, Vienna, Austria).

#### Preprocessing and segmentation

All images –both synthetic and real– are first preprocessed. To remove unwanted background, we subtract the mode of the intensity distribution and zero-clip the resulting image to prevent pixels from having negative values. Then, nuclei visible in the BLUE channel are segmented applying a Laplacian-of-Gaussian filter of size 20 and then thresholding the resulting values above 0.1. Edge objects are removed from the resulting mask.

#### NMF based unmixing

**Figure 2:**
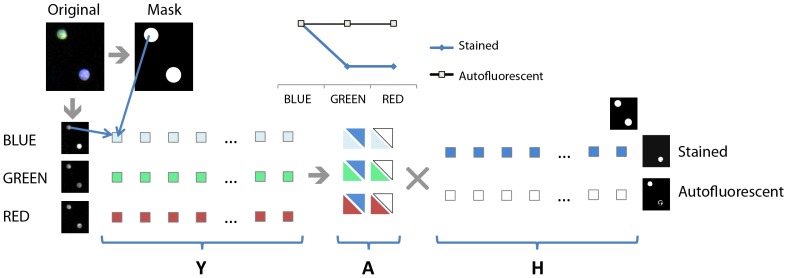
Unmixing approach used for the classification of stained and autofluorescent nuclei. The process begins with the segmentation of the nuclei from the stained –BLUE- channel, creating a mask of all the objects. The pixels of the mask in the corresponding BLUE, GREEN and RED channels are then arranged as the three rows of a single matrix Y. The matrix Y is then decomposed into A and H using NMF-ML. The resulting H matrix rows are used to build two images: the first image will have higher pixel values wherever the original image has a tendency towards blue; the second image will have high pixel values wherever values are similar, i.e. belong to autofluorescent objects. The graph shows a possible initialization matrix for A, before normalization, as used in our experiments. The matrix A components are depicted using a rectangle triangle. The lower part is in the color of the channel, the upper part in the color of the dye.

All pixels belonging to a segmented object are spectrally unmixed. To this end we first build the matrix **Y** from the intensities calculated in the three detection channels, arranged as a *3×N* matrix, where *N* is the total number of pixels in the object. Then, the *3×2* matrix **A** is initialized and the system **Y**≈**AH** solved to calculate the *2×N* fluorochrome emission matrix **H**, using the NMF-ML algorithm described in the previous section. The initialization of matrix **A** is:
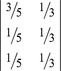



The first column of this matrix has a dominating BLUE component (3/5) that represents the spectrum of stained nuclei. The second column has uniformly distributed values in the three detection channels, approximating the spectrum of autofluorescent nuclei or objects. Note that the sum of each column is one. The unmixing process is graphically shown in Figure 2.

#### Classification

Each segmented object is classified as positive (stained) or autofluorescent nucleus based on a global, per nucleus, measurement performed after spectral unmixing. Namely, it is expected that for a given, properly unmixed pixel, the first spectral component –BLUE- have higher intensity than the other two if that pixel belongs to a stained nucleus, or similar intensity to the other two channels if the pixel belongs to an autofluorescent nucleus. Consequently, to distinguish autofluorescent from stained nuclei, we assign a score to each segmented nucleus according to the number of its pixels where the value of the first (Stained) component is higher than the second (Autofluorescence). We call this score Staining Factor (SF). If the SF is above the threshold calculated during the training phase (see next paragraph), the nucleus is classified as positive (stained), otherwise the nucleus is classified as autofluorescent.

To train the classifier, we calculate both a crosstalk matrix **A** and a threshold for the S using a set of selected images. To calculate **A**, the –three channel– intensities of all pixels of all nuclei segmented from the training image set are arranged as a *3*×*N* matrix **Y**. Then the NMF-ML algorithm is applied to **Y**. The output of the algorithm is a *3*×*2* matrix **A** and a *2*×*N* matrix **H**. For each nucleus we calculate the SF and then manually classify it as either stained or autofluorescent. Since each sampling point in space is represented as a column of **H**, the SF is calculated for each object as the number of points where the value corresponding to that point in the first row of **H** is larger than the value in the second row. The reasoning behind this is that whenever the pixel intensities have a tendency towards the blue side of the spectrum, a higher proportion of points in the object will have a higher value in the first row of **H** than in the second. This results in a high SF value, which in turn indicates positive staining.

To select an appropriate SF threshold **t_SF_**, we calculate the True Positive Rate (TPR) and the False Positive Rate (FPR) for all possible values of **t_SF_**. The TPR or Sensitivity measures the number of correctly identified stained nuclei (true positive) relative to the total number of stained nuclei (true positive plus false negative), while the FPR is defined as the number of autofluorescent nuclei marked as positive (false positive) relative to the total number of autofluorescent nuclei (true negative plus false positive). Among the values of **t_SF_** that result in a TPR higher than 95%, we choose the one having the lowest possible FPR. The reason for this is that in our study, we were more concerned with the possibility of missing a cancer cell than with detecting autofluorescent objects that could be later discarded. Therefore, we fix a minimum TPR ( = recall) of 95%, thus loosing on average only one every 20 cancer cells.

In summary, to classify a nucleus as stained or autofluorescent using the **A** and **t_SF_** found during the training phase, we first build the matrix **Y** for that object and use **A** to calculate **H**; then, we count the number of columns of **H** where the first row has a higher value than the second row (the SF) and classify the object as stained nucleus if the SF value is above **t_SF_** and autofluorescent nucleus otherwise.

### Spectral Unmixing of Multi-labeled Samples

Here our goal is to correctly classify Multi-color Fluorescent In-Situ Hybridization (M-FISH) signals in multiply labeled cells in both synthetic and real images.

#### Synthetic images

Twenty five 3D 3-channel images were generated with two nuclei each one containing two pairs of FISH signals. The nuclei and FISH signals are labeled using fluorochromes emitting in different channels. Each FISH signal was created with a different –random- intensity. The nuclei and the FISH signals were approximated using Gaussian ellipsoids of varying size and eccentricity.

#### Real images: sample preparation and image acquisition

We used two samples: a lung cancer cell line (H460 or H1299) and normal human mononuclear cells obtained from peripheral blood from healthy controls. Nuclei were counterstained with DAPI and labeled with four DNA probes using FISH. Three of the four FISH probes were DNA sequences commonly altered in lung cancer, while the forth was a centromeric probe that is seldom involved in cancer related genetic alterations. Namely, the probes targeted the loci 5p15.2 (labeled with SpectrumGreen), 8q24 (labeled with SpectrumGold) and 7p12 (labeled with SpectrumRed) and the centromere of chromosome 6 (labeled with SpectrumAqua). Seventy three randomly selected nuclei were acquired as image stacks of between 20 and 30 slices thick, with 200 nm inter-slice distance. The objective used was a 63×1.4NA Plan-apochromat oil immersion objective by Zeiss. To acquire the images we used the same microscope and filter cubes described in the previous application with the additional emission filters GREEN (ex. 470/40, dic. 495, em. 525/50) and GOLD (ex. 546/11, dic. 495, em. 575/30). A summary of the excitation and emission bands can be found in Table 1. These filters were chosen to fit the spectra of the five fluorochromes used in our application (see Table 2).

**Table 1:** Filter cubes used in the experiments.

	Filter set name	Ex. band	Dichroic	Em. Band
BLUE	Zeiss Filter set 49 HE	365/50	395	445/50
AQUA	Chroma 31044v2	436/20	455	480/40
GREEN	Zeiss Filter set 09	450–490	510	>515
GOLD	Chroma 41041	546/11	555	575/30
RED	Chroma 41035	546/11	555	605/75

These five sets of filters cover the five different excitation/emission bands needed for the four FISH probes and the DNA counterstain.

**Table 2:** Fluorochromes used and their corresponding filter cubes (see Table 1).

Fluorochrome	Peak ex. (nm)	Peak em. (nm)	Filter cube
Alexa 350	343	442	BLUE
SpectrumAqua	433	480	AQUA
SpectrumGreen	497	524	GREEN
SpectrumGold	530	555	GOLD
SpectrumRed	592	612	RED

The peak excitation and peak emission wavelengths are given for the six fluorochromes used.

Both synthetic and real data can be downloaded from the following ftp site: ftp.codesolorzano.com. Access credentials will be provided upon request by the authors.

#### Preprocessing and segmentation

All images are first corrected for residual chromatic shift using a rigid transformation and deconvolved using the Huygens Scripting deconvolution software (Scientific Volume Imaging, Amsterdam, The Netherlands). Next, FISH signals are segmented using a simple threshold and the resulting mask filtered to eliminate isolated pixels using a morphological erosion [46] with a circle shaped structuring element of radius *r = 2*. We then combine all four binary masks using the OR operator to create a unified binary representation of each nucleus including all its FISH probes.

#### NMF-ML unmixing and measurement of crosstalk

**Figure 3:**
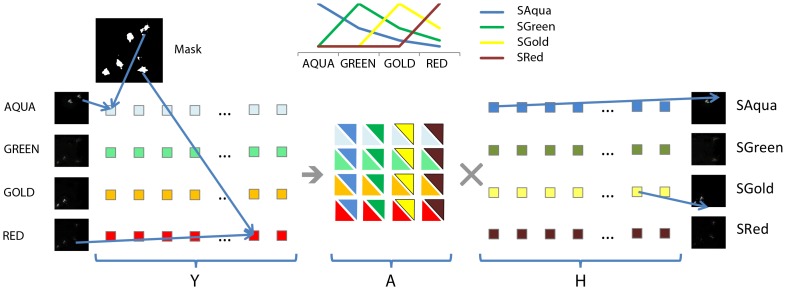
Unmixing approach used to classify Multiple-labeled FISH signals. Four images are acquired in four different channels. Each channel is segmented and the four segmentations combined in a unique mask. The intensities of the pixels are arranged into four rows of a matrix, **Y**. After **A** and **H** are initialized, the unmixing algorithm begins. After convergence, the initial matrix **Y** is decomposed into two matrices **A** and **H**. The columns of the mixing matrix **A** contains the spectra of the four fluorophores as detected in the four channels. The matrix A components are depicted using a rectangle triangle. The lower part is in the color of the channel, the upper part in the color of the dye. The **H** matrix contains the intensity of the four fluorophore emissions at each pixel position and can be used to build four images representing the distribution of the four fluorophores.

We use the unified binary mask to read out the pixels from each of the four channels and arrange them in four rows, thus creating the *4×N* intensity detection matrix **Y**. **A** is then initialized using a *4×4* exponential matrix and the fluorochrome emission matrix **H** (*4×N*) with the detected intensity data (**Y**). The **Y** matrix is then unmixed using our NMF-ML algorithm to recover **A** and **H**. Thus, the four channel input data gets decomposed into four components, each one corresponding to one of the expected fluorescent channels. The resulting **H** matrix is then used to rebuild the four unmixed images, by assigning the intensities in the four channels to a new image at each position of the binary mask (see Figure 3 for a visual representation of the unmixing process).

To quantify the presence of interfering fluorescence into a given fluorescent channel we used a measurement of crosstalk (XT) before and after unmixing. Assuming that we have two filter channels **C_1_** and **C_2_** designed to detect fluorochromes **c_1_** and **c_2_** respectively, the XT of fluorochrome **c_1_** into channel **C_2_** is the mean intensity of the pixels of fluorochrome **c_1_** (those with stronger intensity in **C_1_** than in **C_2_**) in channel **C_2_**, divided by the average intensity of channel **C_2_** for all fluorochromes. This value is 0 if either there are no pixels from fluorochrome **c_1_** in **C_2_** or their intensity is 0.

Our images are three-dimensional, so even after selecting a subset of points with the binary mask, they still contain a considerable amount of data. To speed-up the algorithm, we use the fact that the last step recalculates **H** based solely on **A** and **Y**, discarding any previous estimation of **H: H** ← (**A_c_** ^T^ **A_c_**+ε)^−1^ **A_c_** ^T^ **Y_0_** and setting the negative elements of the matrix to 0. Note that this step is only applicable in images with dark background, such as fluorescent or darkfield images. However it can easily be adapted to brightfield images by working on the inverted image. We can exploit this fact by calculating **A_s_** on a representative subset of the data **Y_s_** and then performing the last step above on the complete data **Y**. We implemented this optimization by selecting the subset of points using the Maximum Intensity Projection of both the input images and the unified binary mask. We build **Y_s_** using the reduced –masked– images, and then apply the NMF-ML algorithm to calculate the **A_s_** and **H_s_** matrices. Next, we recover the **H** matrix and build the **Y** for the 3D image -using the original images and binary mask- and apply the last step of the NMF-ML algorithm. The new **H** can now be used to rebuild the unmixed images, as explained above and shown in Figure 3.

## Results

### Detection of Stained Nuclei in the Presence of Autofluorescence

#### Performance evaluation

To evaluate the performance of our NMF-ML based classification algorithm, we compared it against two other simple approaches: thresholding the stained BLUE channel (as proposed in [8]) or thresholding the color intensity ratio between the first (BLUE) channel and the sum of intensities in all three (BLUE, AQUA, RED) channels. All threshold values have been chosen calculating the TPR and FPR on the training group and selecting -among all threshold values that produce a TPR higher than 95%- the one having the lowest FPR. We used three sets of images: one set was made of synthetic images and two sets of real images. Each set was further divided into a training and a validation group: in the synthetic set, 48 objects were used for training and 336 for validation; in the real image sets, 200 objects were used for training and 461 for validation. For each set, the classifier was trained to obtain the **A** matrix and **t_SF_** values that were used in the validation group. The performance of the classifier is evaluated by calculating the TPR and FPR.

#### Experimental results

To compare the ability of the algorithms to distinguish stained from autofluorescent nuclei, we used the Receiver Operating Characteristic (ROC) curve, which graphically represents the performance of a classifier by showing the TPR and FPR for a range of values of a parameter. In a perfect classifier, all points should be concentrated at the upper left corner (TPR = 1, FPR = 0). A random classifier, on the other hand, tends to stay on the (0,0)-(1,1) diagonal. The Area Under the ROC curve (AUR) is a common way to quantify the performance of a classifier and compare ROC curves.

**Figure 4:**
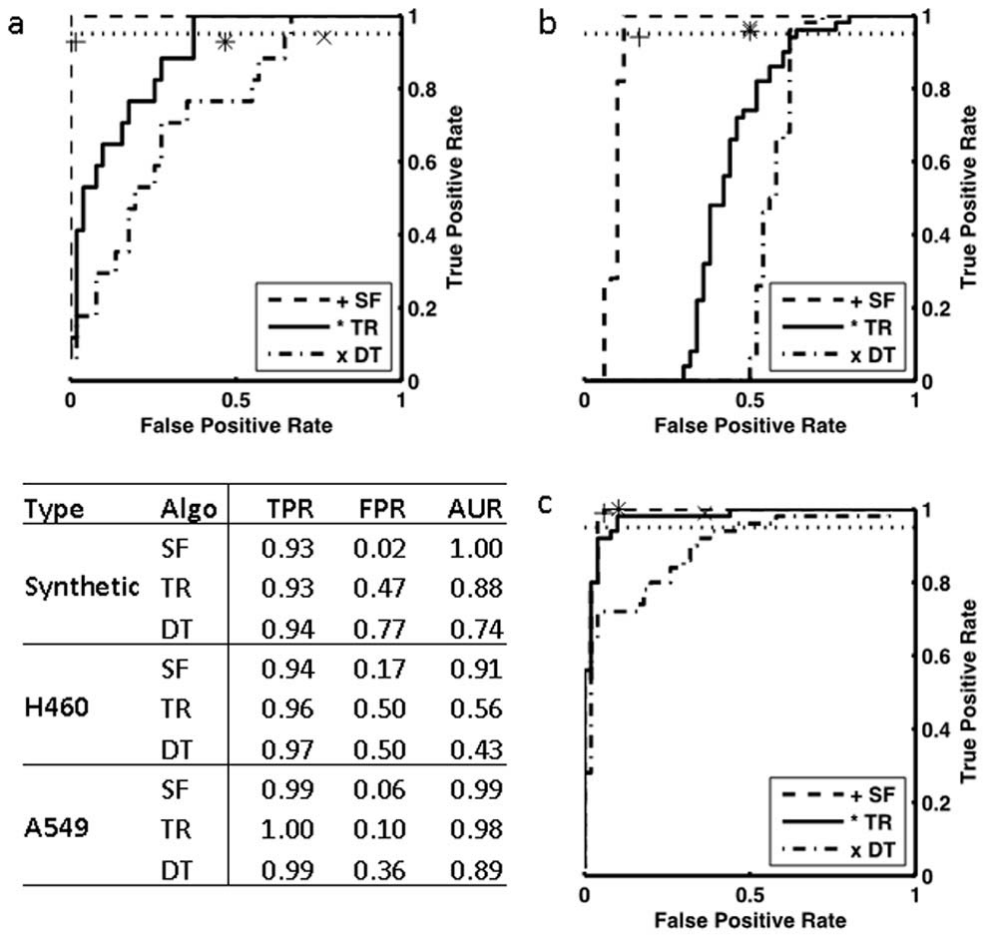
Training and validation of the unmixing algorithm. ROC training curves and validation results for the synthetic dataset (a), the H460 cell line (b) and the A549 cell line (c). SF indicates the data for our algorithm, TR indicates results for the color ratio and DT indicates the results of thresholding the DAPI channel. In the three cases the target true positive ratio at point-of-work was set to 0.95 and can be seen in the graph as a dotted horizontal line. The markers (+,*,x) indicate the performance on the validation data, which is also shown in the table. As an additional performance measure, the table also shows the AUR, the area under the ROC curve, a standard measure to compare classifiers. The results show the benefit of using our algorithm in all three cases.

Figure 4 shows the three ROC curves corresponding to synthetic data (Figure 4a), and to the A549 (Figure 4b) and the H460 (Figure 4c) cell lines along with a table of performance measures. In all three cases, the curve corresponding to our algorithm stays above the other two, suggesting better overall performance. The worst performing classifier is the threshold on the BLUE channel, which supports the observation that a threshold in a single channel is not good enough to distinguish positive from autofluorescent nuclei.

In all the three sample types, AUR is consistently the when using our algorithm. TPR is within a half interval of 0.035 from the target TPR of 0.95, which reflects good consistency between the training and the validation group, given that the threshold chosen using a target TPR on the training set gives a similar TPR in the validation set. The FPR of the three methods varies considerably: our proposed NMF-ML algorithm consistently maintains a low FPR across the sample types (average of 0.08), but the other two show FPR values up to 0.77. Such a high FPR for the target TPR of 0.95 implies that in order to achieve the desired level of sensitivity (TPR) one would have to allow a large number of autofluorescent objects to be misclassified as stained.

### Classification of FISH Signals

#### Algorithm comparison

We used the XT values after unmixing to compare the performance of our NMF-ML algorithm against the NNLS [15] and the NMF-SB [24] algorithms. The convergence criteria have been set to the same value for both NMF algorithms (10^−5^) and both initialized **H** using the detected intensity matrix **Y**. We can use **Y** for initialization because the matrices have the same size and this initialization shows better performance in our results than random components. All the algorithms initialize **A** using the same exponential matrix.

#### Experimental results

The results of the comparison between the three algorithms (our NMF-ML, NNLS and NMF-SB) are presented next from three different points of view.

**Figure 5:**
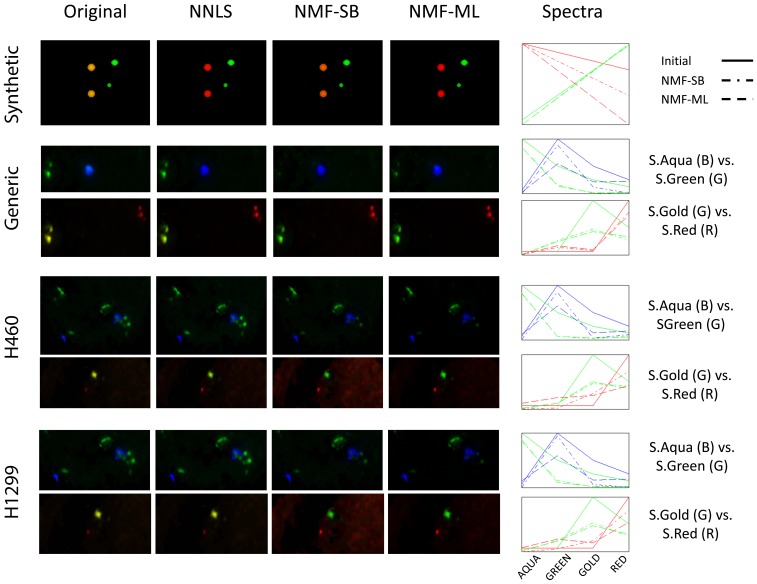
Example of the spectral unmixing of M-FISH samples. The same image is shown across rows. RGB composition of (Top) AQUA channel (R), GREEN channel (G) and GOLD channel (B). (Bottom) GREEN channel (R), GOLD channel (G) and RED channel (B). The last column shows the recovered spectra for the NMF-SB and NMF-ML algorithms, compared with the actual spectrum (synthetic images) or the initial guess (real images).

First, the performance of the unmixing algorithms can be compared visually in Figure 5. The first column shows the original (synthetic or real images) images. The second to fourth columns illustrate results of the unmixing algorithms. The last column shows the recovered spectra for the NMF-SB and NMF-ML algorithms, compared with the actual spectrum (synthetic images) or the initial guess (real images). The synthetic data (top row) exhibits high levels of crosstalk of channel (R) into channel (G), appearing as yellow signals. This is clearly resolved after unmixing with NMF-ML while some residual cross-talk is visible both in the images and the recovered spectrum after unmixing using NNLS and NMF-SB. In the real data, the two emissions with the highest spectral overlap are: SpectrumAqua in the GREEN channel and SpectrumGold in the RED channel. In Figure 5 the control sample is shown in the second row, the H460 cell line in the third row and the H1299 cell line in the bottom row. Each sample contains the RGB composition of the R (AQUA channel), G (GREEN channel) and B (GOLD channel) in the top row and the RGB composition of the R (GREEN channel), G (GOLD channel) and B (RED channel) immediately under. The crosstalk caused by the emission of the SpectrumAqua detected in the GREEN channel is hardly visible in the images. On the other hand, it is clear from the spectra that both NMF methods are able to eliminate the cross-talk existing in the original images and recover both SpectrumAqua and SpectrumGreen emission peaks. The crosstalk caused by the emission of the SpectrumGold fluorochrome detected in the RED channel is seen as Aquamarine color. Some leftover aquamarine can be appreciated in the NNLS results that are practically non-existent in the NMF outputs.

Second, the XT was measured before and after the application of the three algorithms and has been charted for synthetic and real images (Table 3). For the synthetic images, the median (med) XT of the original images is 0.70. The med XT is null for the NMF unmixed images and clearly reduced (med XT = 0.12) for NNLS. For the real images, the med XT of the original images is 0.65. NNLS only achieves a slight reduction (med XT = 0.56), while the improvement using NMF is much larger (NMF SB med XT = 0.12, NMF-ML med XT = 0.04).

**Table 3:** Performance comparison between the three unmixing algorithms (NNLS, NMF-SB and our NMF-ML) for the separation of FISH signals.

	XT		Time	
	Synthetic	Real	Synthetic	Real
Raw	0.70	0.65	N/A	N/A
NNLS	0.12	0.56	0.84″	4′54″
NMF-SB	**0.00**	0.12	1.59″	9′3″
NMF-ML	**0.00**	**0.04**	**0.32″**	**22.19″**

The median crosstalk coefficients (XT) are shown for each algorithm in synthetic and real images, together with the average calculation time for synthetic and real images. The best performer is highlighted in bold. As shown, the calculation time for the NMF-ML algorithm is drastically lower, especially in real images. This difference is mainly thanks to the ability to work on a 2D projection of the 3D stack.

Finally, the results of the statistical test for crosstalk difference are shown in Table 4. NNLS and both NMF algorithms show significant improvement with respect to the unmixed images and both NMF algorithms show a significant improvement over NNLS. When comparing the two NMF algorithms, our NMF-ML shows significantly better results.

**Table 4:** Cross talk reduction of the three compared unmixing methods for both the synthetic and real images.

	NMF-ML	NMF-SB	NNLS	Original
NMF-ML	–	>.999	>.999	>.999
NMF-SB	<0.001	–	>.999	>.999
NNLS	<0.001	<0.001	–	>.999
Original	<0.001	<0.001	<0.001	–

The table shows the p-values obtained when evaluating the average decrease in crosstalk between each pair of unmixing algorithms using a paired one-tailed non-parametric Wilcoxon ranked-sign test. The null-hypothesis in each case is that the change is not significant while the alternative hypothesis is that the algorithm in a given row is significantly worse than the corresponding column algorithm.

As an additional consideration, NMF-ML shows a drastic improvement in computation time when applied to 3D images as shown in Table 3. The average time required to perform the unmixing in biological samples is of around 22 seconds compared to 9 minutes of NMF-SB algorithm and 5 minutes of NNLS.

We also compared the performance (XT) to using the Gauss-based initialization matrix and the results are shown as supplementary material in Figure S1. We observe that the performance of NNLS decreases very significantly (p-value <0.001), when the crosstalk matrix is initialized to a Gaussian instead of an exponential.

## Discussion

The possibility of combining several fluorescent labels is one of the strengths of fluorescence microscopy. However, spectral overlap between fluorochrome emissions limits the number of fluorochromes that can be combined in one sample. A common approach to reduce the spectral overlap or crosstalk combines a full characterization of the fluorochromes -by the acquisition of spectral scans- and the use of analysis algorithms (i.e., LS, NNLS). Spectral scans can be obtained through the use of specialized hardware component to the microscope such as an interferometer, a computer programmed liquid crystal filter (LCTF), an acousto-optic tunable filter (AOTF) or a lambda grating [47]. The use of spectral scanners results in additional costs and therefore, is not always appropriate. A cheaper and simpler alternative is to manually measure the crosstalk coefficients. These measurements need to be repeated for each experiment – or even between acquisitions- and like any manual procedure is prone to error. Blind linear unmixing methods are very helpful by simplifying the experimental setup and avoiding the need for cumbersome spectral characterizations of the fluorochromes. Among others, we have chosen to apply the NMF algorithm because, as opposed to other blind methods, it naturally preserves the positivity of the results. Moreover, NMF can be interpreted as a parts-based representation of the data since only additive combinations are allowed. This correctly reflects the real fluorescent staining of cellular structures.

NMF algorithms are not generally guaranteed to converge. However, it has been shown for Hoyer’s algorithm [39], unpon which NMF-SB is built and which assumes known ratios between the L_1_-norm and the L_2_-norm, that convergence is guaranteed under broad conditions. Using a multi-layer NMF approach (NMF-ML) also considerably improves the uniqueness of the result. Moreover, the implemented multiplicative update rules are enforcing sparseness through the minimization of L1-norm [38]. The remaining main problems of the existing NMF algorithms are their sensitivity to the initialization and their slow convergence. We deal with the former using a crosstalk initialization matrix that takes into account the asymmetric nature of the emission fluorochrome spectra, thus adding robustness to the unmixing process by ensuring proper convergence of the optimization algorithm. Then, we accelerate its convergence by using multi-layer NMF and a regularized pseudo-inverse combined with multiplicative rules.

We have applied the algorithm to the separation of fluorescently stained nuclei from autofluorescent nuclei, and to the spectral separation of Multiple-FISH signals. We tested its performance on both synthetic and real images from biological samples to show that our algorithm provides a good compromise between unmixing performance and speed. In the first application, our method can distinguish stained nuclei from autofluorescence with a much lower FPR -for a similar TPR- than other methods -color ratio and threshold of the stained channel-. The color ratio method assumes that autofluorescent objects emit with the same intensity in all the spectral channels, which is not always a good approximation. The use of a crosstalk matrix adds to the generality of the solution by allowing non-uniform contributions of the various channels. The improved performance of our algorithm suggests that the underlying, more flexible model fits the data better. Additionally, training is used to calculate a threshold for SF designed to exhibit a low FPR and a high TPR. This can be crucial in applications dealing with minimal samples, where the expected percentage of positive objects is very low.

The second application takes advantage of the fact that our method provides both the crosstalk and the fluorochrome emission matrix simultaneously. This eliminates the need for measuring the relative contributions and estimating the mixing matrix beforehand, while still providing excellent unmixing. Moreover, we achieved reliable results even in the presence of strongly overlapping fluorochrome emissions without having to acquire the data with multiple excitation setups.

In summary, the work presented in this paper suggests that NMF-ML is a valuable and practical tool for the blind spectral unmixing of multiply fluorescently stained cellular samples.

## Supporting Information

Figure S1 The charts compare the effects of using a different initialization matrix on the performance of three spectral unmixing algorithms, when applied to the problem of the separation of the signals from four FISH probes (see main article for details). The charted values represent the median of a measure of cross-talk (XT, described in the main article) among all images of the test set: left, the results are shown for 25 synthetic images and right for 73 images from real samples. Both image sets have been tested with two different initialization matrices: the exponential matrix (dark gray, described in the main article) and the Gaussian-based matrix (light gray, proposed by Neher *et al.*). The strongest difference in behavior is shown for NNLS in real images and NMF-SB in synthetic images. The good behavior in the NNLS for real images can be interpreted as a good approximation of the actual crosstalk matrix: the better the approximation, in fact, the lower will the cross-talk be after unmixing. All pairs of datasets corresponding to a different initialization matrix (pairs of light and dark columns) were tested for statistical significance with a paired Wilcoxon rank-test. The significance of the difference is marked with asterisks above each column pair, whenever the difference was in fact significant. The same statistical test was performed for the entire dataset and shows a significant (***) preference for the exponential matrix.
(TIF)
