# Supplementary figures and images for: Efficient Blind Spectral Unmixing of Fluorescently Labeled Samples Using Multi-Layer Non-Negative Matrix Factorization
Source: PLoS One. 2013 Nov 8;8(11):e78504. doi: 10.1371/journal.pone.0078504 (PMC3832632; doi:10.1371/journal.pone.0078504)

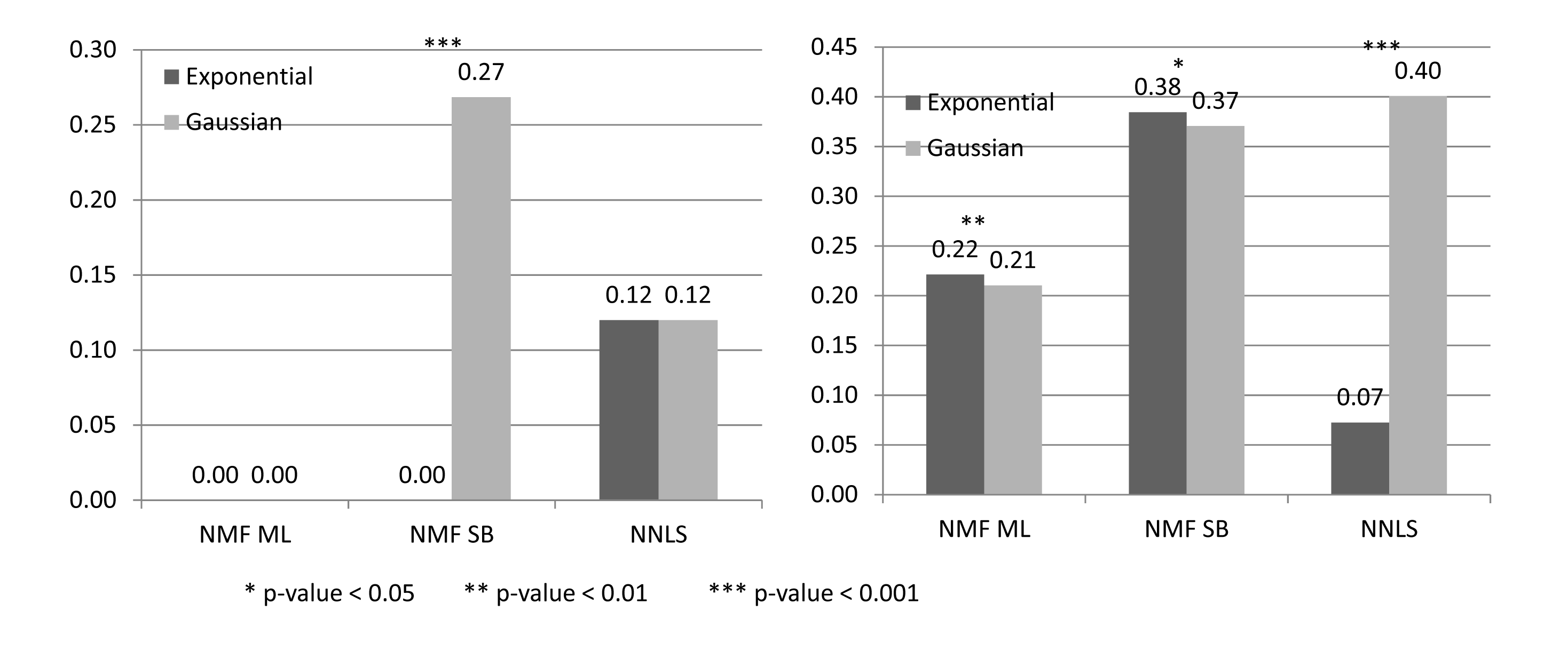

Supplement: Figure S1 — The charts compare the effects of using a different initialization matrix on the performance of three spectral unmixing algorithms, when applied to the problem of the separation of the signals from four FISH probes (see main article for details). The charted values represent the median of a measure of cross-talk (XT, described in the main article) among all images of the test set: left, the results are shown for 25 synthetic images and right for 73 images from real samples. Both image sets have been tested with two different initialization matrices: the exponential matrix (dark gray, described in the main article) and the Gaussian-based matrix (light gray, proposed by Neher et al.). The strongest difference in behavior is shown for NNLS in real images and NMF-SB in synthetic images. The good behavior in the NNLS for real images can be interpreted as a good approximation of the actual crosstalk matrix: the better the approximation, in fact, the lower will the cross-talk be after unmixing. All pairs of datasets corresponding to a different initialization matrix (pairs of light and dark columns) were tested for statistical significance with a paired Wilcoxon rank-test. The significance of the difference is marked with asterisks above each column pair, whenever the difference was in fact significant. The same statistical test was performed for the entire dataset and shows a significant (***) preference for the exponential matrix. (TIF) [file pone.0078504.s001.tif]
